# Impaired cellulose decomposition in a headwater stream receiving subsurface agricultural drainage

**DOI:** 10.1186/s13717-022-00406-9

**Published:** 2022-09-26

**Authors:** Rebecca Poisson, Adam G. Yates

**Affiliations:** 1 Department of Geography, Western University, London, ON Canada; 2 Department of Biology, University of Waterloo, Waterloo University, 200 University Avenue West, Waterloo, ON N2L 3G1 Canada

**Keywords:** Stream, Agriculture, Cotton strip assay, Seasonality, Organic matter breakdown, Benthic respiration

## Abstract

**Background:**

Agricultural development of former wetlands has resulted in many headwater streams being sourced by subsurface agricultural drainage systems. Subsurface drainage inputs can significantly influence stream environmental conditions, such as temperature, hydrology, and water chemistry, that drive ecological function. However, ecological assessments of subsurface drainage impacts are rare. We assessed the impact of an agricultural drainage system on cellulose decomposition and benthic respiration using a paired stream study in a headwater branch of Nissouri Creek, in Ontario, Canada. Adjacent first order segments sourced by a spring-fed marsh and a cropped field with subsurface drainage, as well as the adjoining trunk segment, were sampled over a year using the cotton strip assay to measure cellulose decomposition and benthic respiration.

**Results:**

Assessments of cellulose decomposition revealed a one-third reduction in the drainage-sourced segment compared to marsh-sourced segment. Between segment differences in cellulose decomposition were associated with reduced summer temperatures in the drainage-sourced segment. Impacts of stream cooling from the drainage-sourced segment were transmitted downstream as cellulose decomposition was slower than expected throughout the drainage-sourced segment and for several hundred meters down the adjoining trunk segment. Benthic respiration only differed between the drainage- and marsh-sourced segments in spring, when stream temperatures were similar.

**Conclusions:**

Our findings suggest there may be a widespread reduction in cellulose decomposition in streams across similar agricultural regions where subsurface drainage is prevalent. However, cooling of streams receiving significant amounts of water inputs from subsurface drainage systems may impart increased resiliency to future climate warming.

## Background

Intensive agriculture is a global threat to the biodiversity and ecological function of stream ecosystems (Vörösmarty et al. 2010). Indeed, it has been well documented how agricultural activities such as land clearance, crop cultivation and livestock husbandry impact streams (Allan 2004). However, in addition to land clearance, there has been, and continues to be, significant efforts to enhance food production in regions historically dominated by wetlands through the systematic drainage of excess water from agricultural lands (Blann et al. 2009). For example, it is estimated that in the United States and Canada over 27% and 14% of agricultural lands, respectively, have some form of agricultural drainage system installed (ICID 2018), with implementation at the regional scale being substantially greater (e.g., 85% in Lambton County, Ontario, Canada). Yet, in comparison to most agricultural activities, the potential impacts of these agricultural drainage systems on the ecology of streams are poorly explored.

In wet landscapes agricultural drainage systems are primarily installed as subsurface drainage systems. Subsurface drainage systems typically consist of a series of adjoining plastic, concrete or clay tiles. These tile networks are typically located 0.6 to 1.2 m below the surface and create preferential flow paths that collect and route drainage water to nearby ditches and streams (Blann et al. 2009). The flow regime of drainage tiles is dependent upon regional hydrogeology with more continual discharge occurring in regions where tiles intersect an aquifer (Dolezal et al. 2001). In addition to tile drains, subsurface drainage systems may also include small headwater streams that have been enclosed in a pipe and buried with the aim of increasing rate of flow of water from cultivated lands (Stammler et al. 2013). Combined, tile drains and buried streams can account for a significant portion of the headwater network in agricultural regions (Stammler et al. 2013), and greatly change physicochemical conditions of receiving channels downstream (Blann et al. 2009).

Subsurface drainage systems have been shown to impact hydrologic, nutrient and thermal regimes of receiving streams (reviewed by Blann et al. 2009). For example, streams receiving water from subsurface drainage systems have been found to have greater baseflow, total annual flow and minimum annual flow (e.g., Schilling and Libra 2003; Schilling and Helmers 2008). Likewise, subsurface drainage water has been linked to nutrient enrichment of streams as a result of increased delivery of dissolved forms of nitrogen and phosphorus from croplands (King et al. 2015; Royer et al. 2006; Williams et al. 2015). In addition, drainage water from drainage systems has been found to have increased amounts of agricultural contaminants, such as pesticides (Kladivko et al. 2001). Less recognized in the literature is that subsurface drainage water may impact stream thermal regimes as subsurface drainage water may be thermally distinct from surface waters, particularly from fields where tiles drain shallow groundwater aquifers. Thus, the increased delivery of cool, shallow groundwater to streams may moderate daily and seasonal temperature fluctuations. Together these physicochemical alterations are likely to influence stream biota and the ecosystem functions they perform (Blann et al. 2009). In particular, subsurface drainage systems may impact carbon cycling in headwater streams as it has been well demonstrated that breakdown of coarse particulate organic matter by heterotrophic microbial communities is strongly regulated by a stream’s hydrologic, nutrient and thermal status (Tank et al. 2010; Young et al. 2008). However, to date there has been little study of the potential impacts of subsurface drainage systems on organic matter breakdown and stream function more broadly.

The goal of our study was to increase understanding of how subsurface agricultural drainage systems may impact stream ecosystem function. We addressed this goal by conducting an assessment of cellulose decomposition and benthic respiration using a paired stream study in a headwater branch of Nissouri Creek, located in southwestern Ontario, Canada. The headwater branch consisted of two first order segments originating from either a natural spring-fed wetland or a cropped field drained by a subsurface drainage system, as well as the adjoining trunk segment. Stream sampling was conducted throughout a calendar year to: (1) compare temporal patterns in cellulose decomposition and benthic respiration over the course of a year; (2) compare cellulose decomposition among and within stream segments and assess if the outcome of the comparison was dependent upon season, and; (3) determine the likely environmental drivers of among segment differences in cellulose decomposition.

## Methods

### Study area

**Fig. 1:**
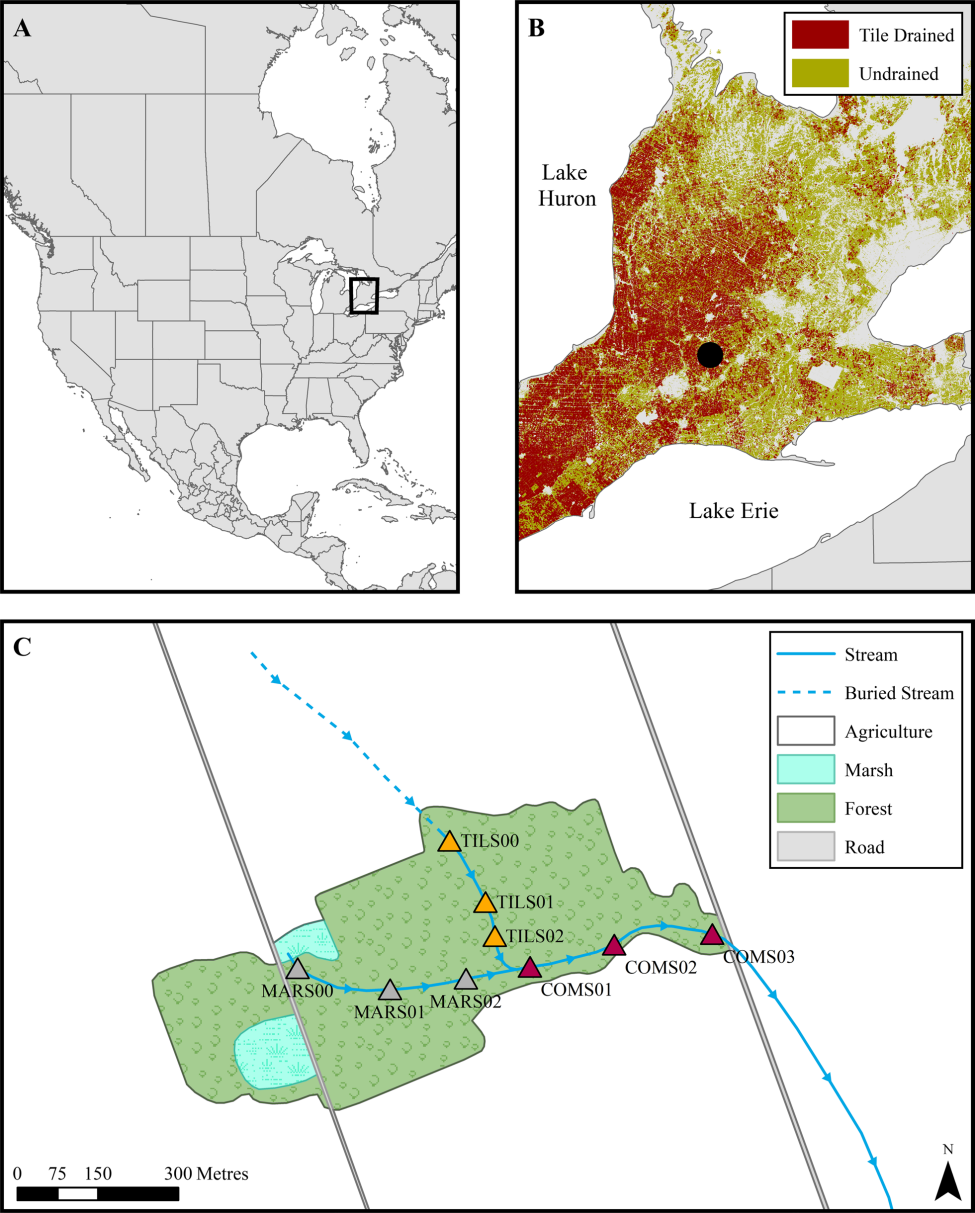
Locations of study area (open square) in the Great Lakes region of North America (**A**) and Nissouri Creek subwatershed (closed circle) in the agriculturally dominated landscape of southwestern Ontario (**B**), where the majority of agricultural lands have tile drainage (red shading). Panel **C** shows the studied headwater branch of Nissouri Creek located in a woodlot with stream sampling sites distributed along two first order segments draining a spring-fed marsh (western branch, grey triangles) and a tile drained field (northern branch, orange triangles), as well as the downstream combined segment (magenta triangles). Blue arrows on stream lines indicate direction of flow

Our study was conducted in a small, headwater stream network located in southwestern Ontario, Canada (Fig. 1). Southwestern Ontario experiences a humid continental climate, due to proximity to Laurentian Great Lakes, with temperatures averaging 27 °C in July and − 10 °C in January (Stratford Station; Government of Canada 2021). The average annual precipitation of this region is approximately 1025 mm (Stratford Station; Government of Canada 2021). The geology of southwestern Ontario is dominated by calcareous Paleozoic bedrock with deep, nutrient rich soils derived from glacial till, outwash and lake plains. Historically, the region’s land cover consisted primarily of upland deciduous forest and treed wetlands (Butt et al. 2005). Following European settlement the majority of the forest was cleared for agricultural activities, leading to the agriculturally dominated (approximately 72% agricultural cover) landscape present today. Moreover, significant hydrologic modification occurred across the region to drain wetlands and improve arability. Wetland drainage often involved the installation of subsurface tile drain systems as well as burial of low order streams. These practices continue to occur and to date have led to about 45% of agricultural lands being tile drained and an estimated 14% of total stream length being buried (Kokulan 2019; Stammler et al. 2013). Thus, many streams in Southern Ontario that historically drained groundwater fed wetlands are now sourced by agricultural drainage systems collecting water beneath cropland.

Within the southwestern Ontario region we selected the headwaters of the Nissouri Creek watershed to assess the potential impact of subsurface drainage systems on stream ecosystem functions (Fig. 1). The 31 km^2^ Nissouri Creek watershed is emblematic of small watersheds in southwestern Ontario. Stream channels are sourced by groundwater flow that historically would have been associated with wetland areas where the water table was near the surface. Moreover, land cover is now dominated by agricultural fields (86%) with remnant patches of forest (12%) and wetlands (1%) comprising the remaining lands (Ontario Ministry of the Environment 2012). Crop cultivation in Nissouri Creek’s watershed consists primarily of corn (40%) with some forage and fodder crops (12%), soybean (10%), and grains (5%) while livestock consists primarily of cattle, poultry and pigs (Ontario Ministry of the Environment 2012). Tile drainage systems are prevalent throughout the watershed (approximately 60% of agricultural fields) and significant sections of the headwater network have been buried.

In a landscape where subsurface drainage systems are prevalent, a headwater branch of Nissouri Creek provided a unique opportunity to assess the potential impact an agricultural drainage system may have on stream ecosystem function. This branch of Nissouri Creek originates in two first order stream segments, the westernmost segment (hereafter marsh segment) originates in a spring-fed marsh, buffered from the surrounding agricultural lands and roadway by abandoned pasture (Fig. 1). In contrast, the eastern segment (hereafter tile segment) now originates in a 20 ha agricultural field (crop rotation: alfalfa, corn, soybeans) with subsurface drainage where the upper 100 m of the stream channel was buried several decades ago. Both the wetland and tile segments flow a distance of approximately 360 m and 250 m, respectively, through a 22 ha woodlot comprised primarily of Eastern White Cedar (*Thuja occidentalis*) and Yellow Birch (*Betula alleghaniensis*) before combining in a second order stream (hereafter combined segment) that flows an additional 400 m before exiting the woodlot.

Within the woodlot nine sampling sites were established along the stream network (Fig. 1), with 3 sites along each stream segment (marsh segment: MARS00, MARS01, MARS02; tile segment: TILS00, TILS01, TILS02; combined segment: COMS01, COMS02, COMS03). MARS00, TILS00, COMS01 were located within 10 m of the initiation of open channel in their respective segments. MARS01, TILS01, and COMS02 were located in the middle of their respective branches (approx. 180 m, 125 m, and 165 m from source, respectively). MARS02, TILS02 and COMS03 were located near the end of their respective branches (approx. 325 m, 195 m, and 365 m from source, respectively); with MARS02 and TILS02 located approximately 50 m upstream of where the branches adjoin. All sampling sites were shaded by extensive forest canopy cover, however, channel widths and depths varied (approximately 30 to 150 cm and 3 to 15 cm, respectively) among sites. Substrate was dominated by sand at five of the sites (MARS01, TILS01, TILS02, COMS02, COMS03). In contrast, MARS00 and MARS02 were silt-dominated and gravel dominated the substrate at COMS01 and TILS00.

All nine sites were sampled once per season to assess if cellulose decomposition differed among the stream segments and if that difference varied with position in the network and/or season. The summer sampling period took place between July 23, 2020 and August 19, 2020; autumn sampling between October 13, 2020 and November 9, 2020; winter sampling from January 27, 2021 to March 9, 2021, and; spring sampling between April 14, 2021 and May 20, 2021. Samples for site MARS00 during the autumn are missing due to vandalism.

In addition to seasonal sampling, temporal patterns of cellulose decomposition and benthic respiration were assessed and compared at the middle position of each stream segment (i.e., MARS01, TILS01, COMS02; Fig. 1). Cellulose decomposition and benthic respiration was sampled at regular intervals (average 4 weeks) at each of these sites from May 27, 2020 through to June 16, 2021. Middle position sites were comparable in bank full and wetted widths, depth, velocity, canopy cover and substrate (Table 1).

**Table 1:** Physical characteristics of stream reaches used for the temporal assessment of impacts of subsurface drainage on cellulose decomposition and benthic respiration in a forested network in Southern Ontario, Canada

Site	Width (m)	Depth (cm)	Velocity (m/s)	Canopy cover	Substrate
MARS01	0.884	4.996	0.0114	Full	Sand
TILS01	1.142	3.240	0.0728	Full	Sand
COMS02	1.136	4.516	0.0662	Full	Sand

Measurements are averages for the study reaches and were collected in the summer of 2020

### Field and Laboratory Procedures

Cellulose decomposition was estimated using the cotton strip assay (CSA; Tiegs et al. 2013), where loss of tensile strength in cotton strips is used as a standardized, surrogate measure of organic matter breakdown. Preparation, deployment, retrieval and processing of the cotton strips followed procedures in Tiegs et al. (2013). In brief, six cotton strips (2.5 cm by 8 cm with 3 mm length frayed edges) cut from Fredix-brand unprimed 12-oz. heavyweight cotton fabric, Style #548 (Fredrix, Lawrenceville, GA, USA), were attached to a piece of chain using cable binders at approximately 30 cm intervals. The chain was anchored to the streambed in a riffle habitat of each site using rebar. Strips were incubated for approximately three to six weeks, depending on the season, to achieve an average tensile loss of 50%. Following incubation, the strips were retrieved and sterilized in 70% ethanol to inhibit further decomposition and transferred to the lab.

In the lab, cotton strips were dried at 40 °C for 24 h before being assessed for tensile strength loss. Tensile strength was measured using a tensiometer and test stand (Force Gauge, Model M3-100) with a pull rate of 2 cm/min until peak tension was achieved. Tensile strength in treated strips was compared to the mean of 50 reference strips that underwent the same processes, but were incubated in distilled water. Loss of tensile strength, used to assess the rate of OM breakdown, was calculated using Eq. (1).

1 $${\text{Tensile loss}}\left( \% \right) = 1 - \left[ {\left( {\frac{{{\text{Tensile}}{\mkern 1mu}\,{\text{Strength}}_{{{\text{Treatment}}}} }}{{{\text{Tensile}}{\mkern 1mu}\,{\text{Strength}}_{{{\text{Reference}}}} }} \times {\text{100}}} \right)} \right] \div \;{\text{Incubation}}{\mkern 1mu} \,{\text{time}}$$

where Tensile Strength_Treatment_ is the maximum tensile strength of each of the strips incubated in the field, and Tensile Strength_Reference_ is the mean maximum tensile strength of 50 strips that were not incubated in the field but otherwise went through the same process.

Measurements of respiration were collected following procedure in Tiegs et al. (2013) in all seasons except winter due to limitations with the oxygen sensors in sub-zero temperatures. At each site, six, 200 mL, dark chambers (3 control chambers and 3 chambers containing 2 strips each) were completely filled with streamwater, capped, and placed on the streambed for 2 h. Dissolved oxygen (DO) was measured using an Ultrapen (Model PT5, Myron L Company) before and after the 2-h incubation. Upon removal from the chambers, strips were sterilized for 30 s in ethanol and then taken to the lab to be dried at 40 °C for 24 h. Strip respiration was calculated using Eq. (2) modified from Tiegs et al. (2013).

2 $${R}_{\mathrm{strip}}= \frac{\left[\left({\mathrm{DO}}_{\mathrm{Stream}}- {\mathrm{DO}}_{\mathrm{strip post}}\right)- \left({\mathrm{DO}}_{\mathrm{Stream}}- {\mathrm{DO}}_{\mathrm{control post}}\right)\right]}{[\left({\mathrm{VH}}_{2}\mathrm{O Chamber}\left(L\right)\right)\times t]}$$

where DO_Stream_ is the DO concentration in the streamwater at the start of the 2 h incubation period, DO_strip post_ is the DO concentration in the chamber with the cotton strips after the incubation period, DO_control_ is the DO concentration in the control chamber after the incubation period, VH_2_O Chamber is the volume of water in a respiration chamber, and *t* is the duration of the incubation.

For each sampling event water temperature and water chemistry were assessed at each of the nine sites. Water temperature was measured every hour using HOBO loggers (UA-002-64, Onset) deployed at the same locations and time intervals as the cotton strips. Average daily temperatures, as well as average daily minimum and maximum temperatures were calculated for each day. Total degree days was also calculated following Benfield (2006). Dominant bioavailable nutrient forms (i.e., dissolved organic carbon (DOC), nitrate-nitrite (${\mathrm{NO}}_{3}^{-}$ + ${\mathrm{NO}}_{2}^{-}$), soluble reactive phosphorus (SRP)) were determined from grab water samples collected in a turbulent area of each site. Samples were analyzed for DOC, using a Total Organic Carbon Analyzer (detection limit of 0.1 mg C/L) and nitrate-nitrite and SRP, using an Automated Ion Analyzer (detection limit of 1 μg N/L and 2 μg P/L for nitrate-nitrite and SRP, respectively). Instantaneous measures of specific conductivity (SPC) and pH were collected using a handheld multi-parameter sonde (YSI, Professional Plus).

Point measures of velocity and depth were also collected at each cotton strip deployed at each of the nine sites during the seasonal sampling events. Depth was measured using a metre stick whereas as velocity was measure at the bed using a OTT MF Pro water flow meter.

Stream stage was recorded every 30 min over the duration of the study using level loggers (U20-001-04, Onset) at the three middle sites. Level loggers were strapped to angle iron deployed in the deepest pool present at each of the sites. Depth measurements were taken at the logger on each sampling event to validate measures of stage. Collected stage data was also corrected for changes in atmospheric barometric pressure using a fourth level logger deployed at the edge of the woodlot.

### Data analysis

To examine temporal patterns of cellulose decomposition, benthic respiration and environmental characteristics, time-series plots were generated depending upon the type of data that was available. For data types that were measured as a snapshot of current conditions (i.e., water chemistry, cellulose decomposition, benthic respiration), measured values were assigned to the sampling event. For data types that were measured continuously (i.e., water level, stream temperature), values were the average of the daily means over the sampling period. Time-series plots were visually analyzed to assess trends through time.

In addition to visual analysis, separate generalized linear mixed-effects models (GLMM) were conducted in TIBCO Statistica (version 13.5) to assess differences in cellulose decomposition and benthic respiration (*α* = 0.05). Factors in both models were site (levels: MARS01, TILS01, COMS02) and sampling date, as well as their interaction (Site × Date). The GLMM for cellulose decomposition had 15 sampling dates, whereas there were 9 sampling dates for benthic respiration. Degree days and average daily water level for each site and sampling period was also added to the model as covariates. Strip was included as a random variable to account for variation associated with individual cotton strips within each site and sampling event. In cases where a significant Site × Date interaction term was found, post-hoc general linear models (GLMs; *α* = 0.05) were completed to compare the measures of cellulose decomposition and benthic respiration among sites for each individual sampling date. Significant GLMs were followed by a pairwise Tukey’s test to determine which sites differed (*α* = 0.05).

A GLMM was also used to assess spatio-temporal differences in cellulose decomposition among the nine sites across the four seasons (*α* = 0.05). Factors in the model were segment (levels: Marsh, Tile, Combined), position (upper, middle, lower) and season (summer, autumn, winter, spring). A nested model was used where fixed effects were season and segment as well as their interaction (season x segment), and position (nested in segment and season). Strip was added as a random variable. The GLMM analysis was performed in TIBCO Statistica (version 13.5).

Partial least squares (PLS) regression was used to weigh the importance of hydrology (i.e., velocity, depth), stream temperature (i.e., degree day/day, average daily temperature range), and water chemistry (i.e., DOC, nitrate-nitrite, SRP, pH, SPC) variables on cellulose decomposition. For data with snapshot measurements (i.e., depth, velocity, water chemistry, tensile loss), individual values were assigned to the respective sampling period. For data types with continuous measurements (i.e., stream temperature), values were summarized over the sampling period. Specifically, degree day/day was calculated by totaling the average daily temperatures for each incubation period and dividing it by the incubation time, in days. Moreover, average daily stream temperature range was calculated by averaging mean daily stream temperature ranges over each incubation period. All predictor variables were normalized prior to analysis to account for differences in units. The goodness of prediction fit (*Q*^2^), which compares the observed values to the predicted values, was used to evaluate model performance (*Q*^2^ > 0.097). To evaluate the total explanatory capacity of the model, the sum of each component’s explanatory capacity (*R*_*Y*_^2^) was calculated and only components that explained more than 10% of the variation of tensile loss were retained. The influence of each factor was assessed using variable importance on the projection (VIP) scores and only factors with VIP scores greater than 1.0 were considered important for explaining tensile loss. *X* scores of the significant variables were examined to determine the direction of association. PLS regression was performed in TIBCO Statistica (version 13.5).

## Results

### Temporal patterns in cellulose decomposition

**Fig. 2:**
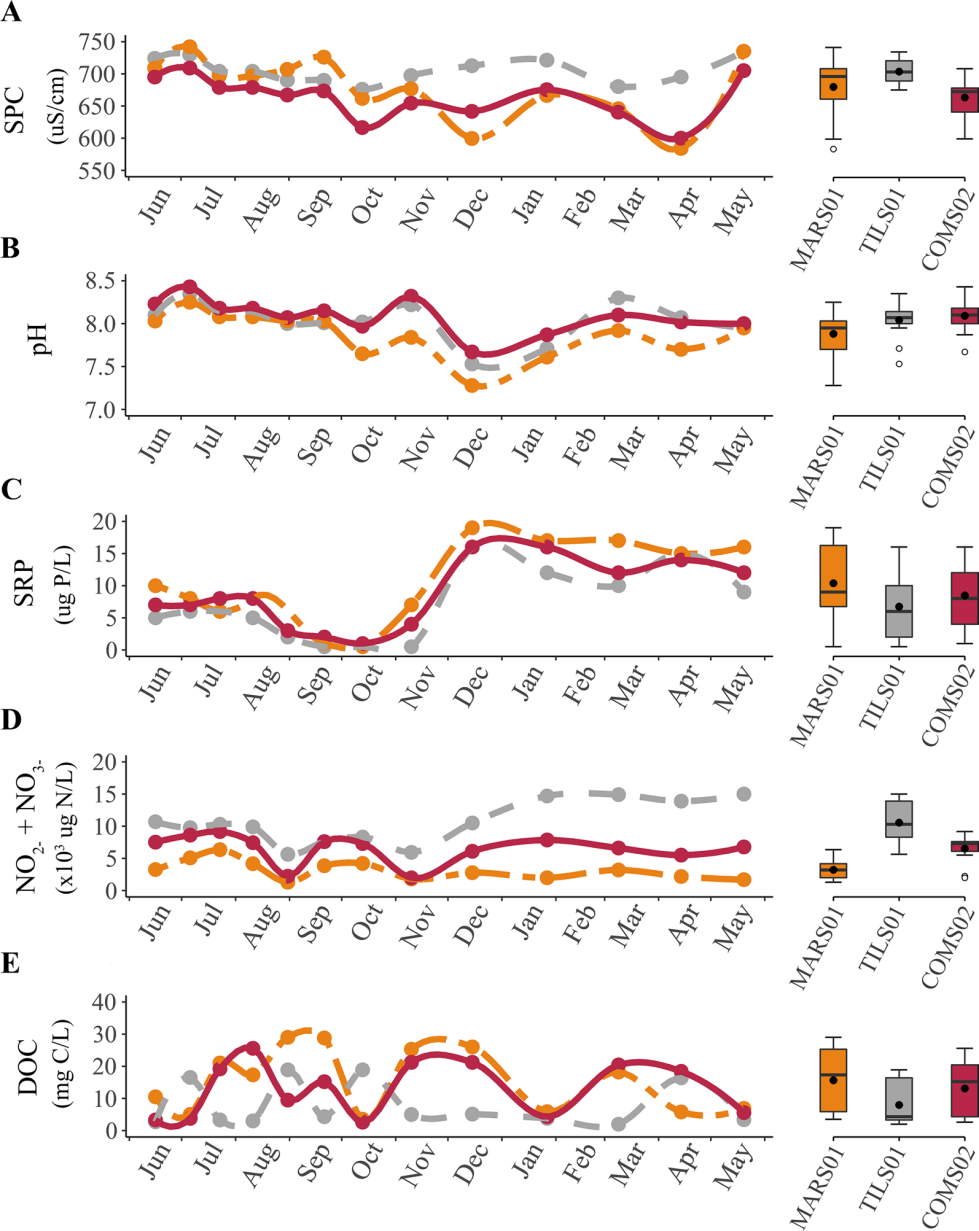
Time series of specific conductivity (SPC) (**A**), pH (**B**), soluble reactive phosphorus (SRP) (**C**), nitrate-nitrite (**D**), and dissolved organic carbon (DOC) (**E**) with boxplots (25th, 50th, and 75th percentiles; whiskers denote ± 1.5 × interquartile range; black dots denote mean) for sites in segments sourced by a marsh (two dash, orange) and tile drain (dash, grey), as well as the downstream combined segment (solid, magenta) over 13 sampling events from June 2020 to May 2021. Closed circles denote each sampling event

SPC was, on average, greater at TILS01 than at COMS02 (*p* = 0.017), but mean SPC at MARS01 did not differ from TILS01 or COMS02 (*p* = 0.21 and *p* = 0.48, respectively; Fig. 2a). From June into October, all stream sites exhibited SPC values within 50 µS/cm, whereas from November into April measured values at TILS01 were at least 50 µS/cm, and sometimes more than 100 µS/cm, greater than those measured at MARS01 and COMS02. In contrast, average pH did not differ statistically among the three sites (*p* = 0.06) and indeed, measured values for the sites only differed by more than 0.2 in autumn and spring (Fig. 2b).

Mean SRP did not differ among the three sites (*p* = 0.15; Fig. 2c). Moreover, for all stream sites, SRP was between 5 and 10 µg P/L in summer, near or below detection in autumn (i.e., 1 µg P/L) and between 10 and 20 µg P/L in winter. In contrast, nitrate-nitrite was different among all sites (*p* < 0.001) as mean concentrations were at least 160% and as much as 330% greater at TILS01 than at COMS02 and MARS01 (Fig. 2d). Among site variation in site nitrate-nitrite averages were driven by winter and spring samples as opposed to summer and autumn. Unlike SRP and nitrate-nitrite, DOC did not show a clear seasonal pattern at any of the sampled stream sites and although mean DOC concentrations were approximately twice as large at COMS02 and MARS01 as at TILS01 the differences were not statistically significant (*p* = 0.07; Fig. 2e).

**Fig. 3:**
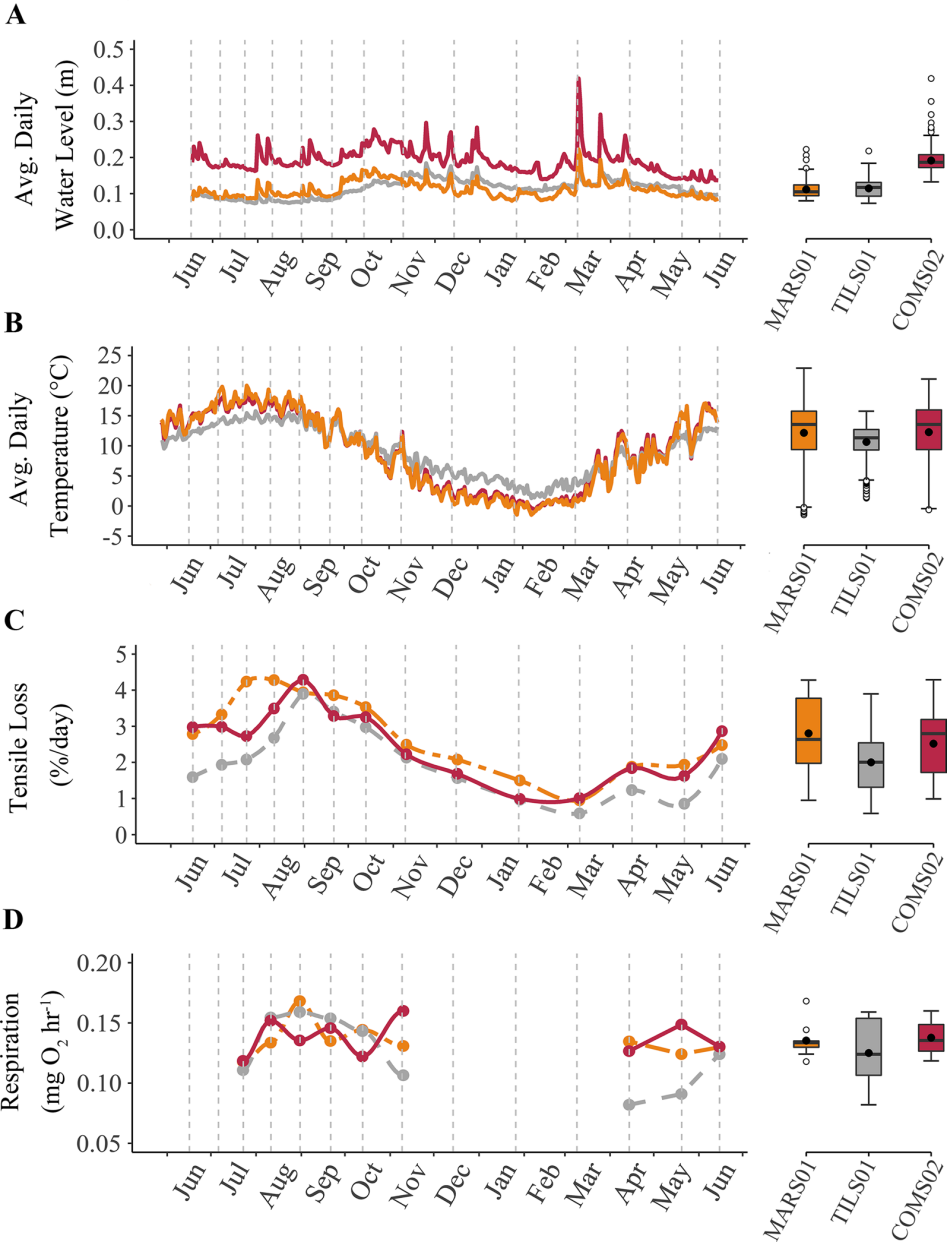
Time series and boxplots of average daily water level (**A**), average daily stream temperature (**B**), % tensile loss per day (**C**), and benthic respiration (**D**) segments sourced by the marsh (two dash, orange) and tile drain (dash, grey), as well as the downstream combined segment (solid, magenta) of a headwater stream branch in southwestern Ontario, Canada sampled from June 2020 through June 2021. Grey, vertical, dashed lines denote sampling events. Boxplots indicate 25th, 50th, and 75th percentiles (box), ± 1.5 × interquartile range (whiskers), outliers (open circles) and mean (closed circles)

Differences in mean daily water level were less than 0.01 m between MARS01 and TILS01 (*p* = 0.48), but were, on average, twice as high in COMS02 as (*p*’s < 0.001; Fig. 3a). An increase in mean water level was apparent in all stream sites from the summer to fall season. Afterwards, mean water levels decreased from the fall to the winter season and remained similar into the spring season.

Mean daily average streamwater temperatures differed by less than half a degree between MARS01, TILS01 and COMS02 throughout the study year (*p* = 0.69; Fig. 3b). Stream temperature for all sites steadily increased from June through September before gradually decreasing in February, then increased in April to May. However, water at TILS01 was, on average, about 3 °C colder from July to August and about 3 °C warmer from December to February, in comparison to MARS01 and COMS02.

Averaged over the year, MARS01 had the greatest mean tensile loss (2.83 ± 1.11%/day), followed by COMS02 (2.49 ± 1.01%/day), and TILS01 (1.99 ± 1.01%/day; Fig. 3c). Tensile loss for all stream sites steadily increased to a maximum from June to September before gradually decreasing to a minimum in early March, then increased in April before declining in May. The GLMM assessing temporal patterns in cellulose decomposition among the three sites showed a significant site by date interaction term (*p* < 0.001), as well as a significant effect of the covariate, degree days (*p* = 0.01). The water level covariate was not significant (*p* > 0.05). Assessment of residuals showed no evidence of non-linearities in the data.

Subsequent GLMs testing among site differences in tensile loss for each sampling period indicated that differences occurred in all but three periods (*p* < 0.05). In the periods inclusive of May 27 to July 27 tensile loss was between 50 and 35% slower at TILS01 than MARS01 and COMS02 (*p*’s < 0.005). Tensile loss was also slower at TILS01 than at the other two sites in the July 27 to August 11 period (*p*’s < 0.013), by 16% and 7% for the MARS01 and COMS02, respectively. MARS01 and COMS02 also differed (*p* = 0.016) in this mid-summer period, with tensile loss being slower at COMS02. In contrast, there was no difference in tensile loss among the three sites in the Aug 11 to Aug 31 period (*p* = 0.13). Tensile loss in the August 31 to September period at MARS01 was 12% and 15% faster than at TILS01 (*p* = 0.045) and COMS02 (*p* = 0.01), respectively, before not differing among any of the sites from September 21 to November 10. MARS01 also exhibited about 20 to 35% more rapid tensile loss than the other two sites for the November 9 to December 15 (*p*’s ≤ 0.002) and December 15 to January 27 (*p*’s < 0.001) periods. However, there was no difference between MARS01 and COMS02 for the periods inclusive of January 27 through May 20, although both sites had at least 35% faster tensile loss than TILS01 (*p*’s ≤ 0.002). Only TILS01 and COMS02 differed (*p* = 0.003) in the May 20 to June 16 period, with tensile loss being 27% slower at TILS01.

Averaged over the study year, COMS02 had the greatest mean respiration (0.138 ± 0.015 mg O_2_ h^−1^), followed by MARS01 (0.136 ± 0.015 mg O_2_ h^−1^), and TILS01 (0.125 ± 0.031 mg O_2_ h^−1^; Fig. 3d). Unlike tensile loss, respiration did not exhibit a clear seasonal trend at any of the stream sites. However, results of the GLMM assessing benthic respiration showed a significant site × date interaction term (*p* = 0.024). The degree day and water level covariate terms were not significant (*p*’s ≥ 0.24). GLM’s comparing benthic respiration among the three sites for each sampling date had *p*-values greater than 0.05 for six of the nine sampling events. Exceptions were the autumn period of October 13 to November 10 (*p* = 0.031) and the spring periods March 9 to April 15 (*p* = 0.037) and April 15 to May 20 (*p* = 0.009). For the autumn period, pairwise comparisons indicated that benthic respiration was greater at COMS02 than at TILS01 (*p* = 0.026), but rates of respiration at MARS01 did not differ from those at either COMS02 (*p* = 0.20) or TILS01 (*p* = 0.30). Benthic respiration at TILS01 also differed from the other sites in the two spring sampling events, particularly if *p*-values below 0.1 are considered, which is reasonable given the small sample size per site (*n* = 3). For the March 9 to April 15 period, respiration at TILS01 was on average about 60% of that at MARS01 (*p* = 0.042) and 65% that at COMS02 (*p* = 0.077). The magnitude of difference in respiration was similar for the April 15 to May 20 period although in this period respiration at TILS01 was on average 61% of that at COMS02 (*p* = 0.007) and 75% of that at MARS01 (*p* = 0.076).

### Spatio-temporal patterns of cellulose decomposition

**Fig. 4:**
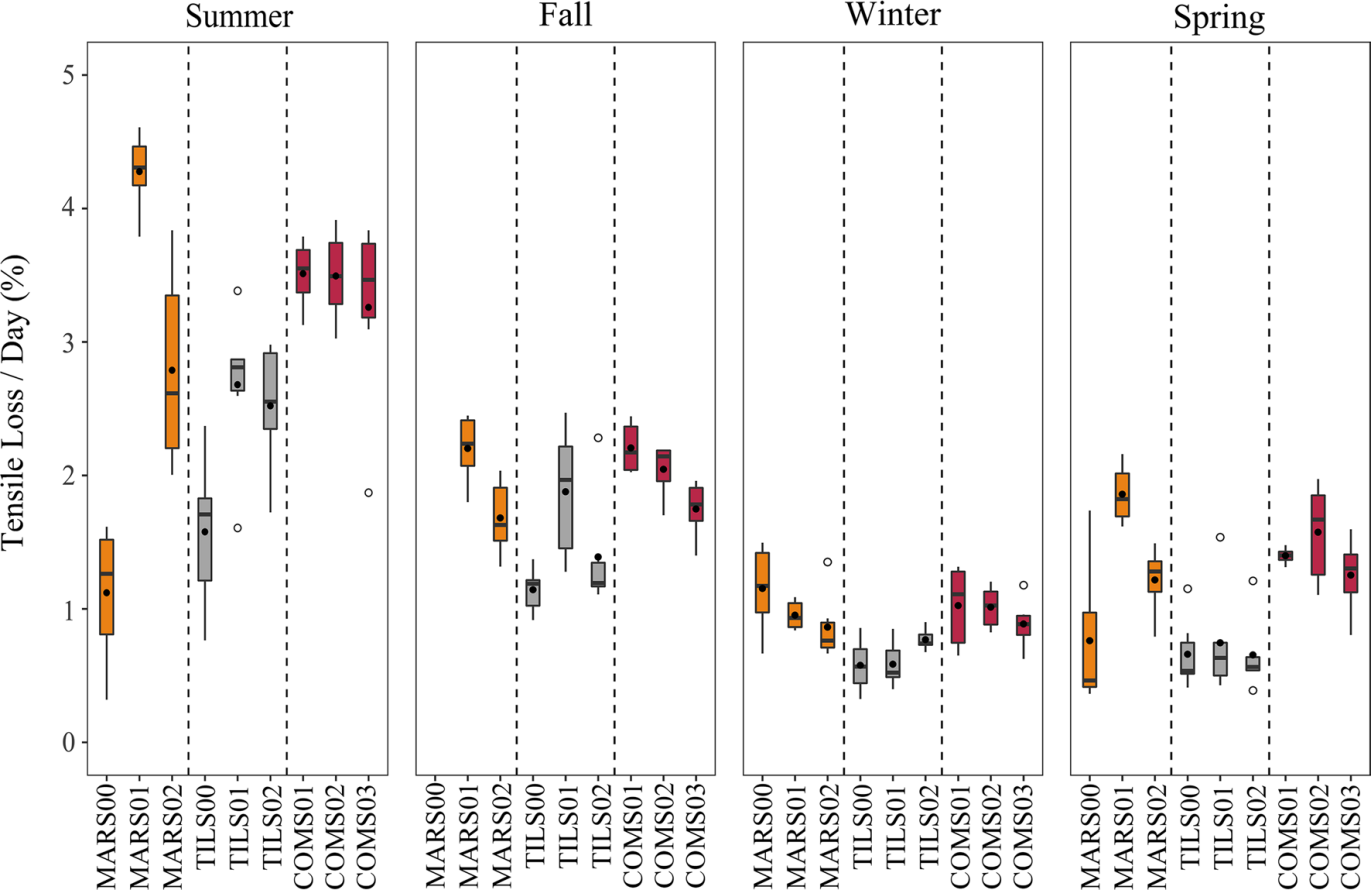
Boxplots summarizing % tensile loss per day at three sites located at the top, middle, and bottom positions of three segments (marsh sourced (orange), tile-drain sourced (grey), and combined sourced (magenta)) comprising a headwater stream branch in southwestern Ontario, Canada sampled in summer and fall of 2020 as well as winter and spring of 2021. Boxplots indicate 25th, 50th, and 75th percentiles (box), ± 1.5 × interquartile range (whiskers), outliers (open circles) and mean (closed circles)

Mean tensile loss among all cotton strips was 1.64 ± 1.0%/day. The GLMM assessing spatio-temporal differences in tensile loss indicated that season, stream segment and position factors, as well as the season by location interactions, were all significant (*p* < 0.001). Tensile loss was on average greater in the marsh and combined segments than the tile segment for all seasons (Fig. 4). However, the differences among segments were dependent upon the position as summer mean tensile loss at the uppermost site of the marsh segment was less than that of any other site. Moreover, the two uppermost positions on the marsh and tile segments had the lowest mean tensile loss except in winter when mean tensile loss was largest at the uppermost marsh and similar at the upper and middle sites of the tile segment. Likewise, all tile segment sites were similar in mean tensile loss in spring and smaller than all sites on the other two segments excepting the upper marsh site. Within segment patterns for the marsh segment as mean tensile loss was typically smallest in the uppermost site and greatest at the middle site, except in winter when tensile loss declined from upstream to downstream. The tile segment had the same longitudinal pattern to the marsh segment in summer and autumn, but exhibited little longitudinal variation in winter and spring. The combined segment generally had declining tensile loss from upstream to downstream although differences among positions were typically small (< 0.5%/day) in all seasons.

**Fig. 5:**
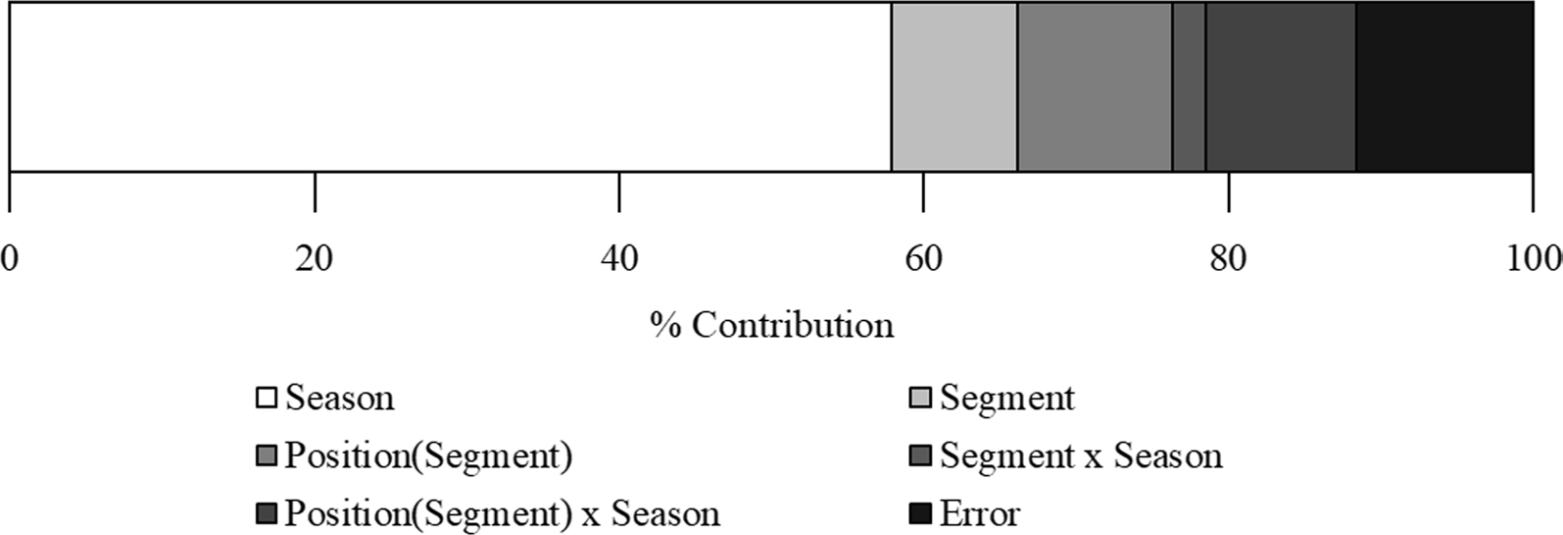
Percent contribution of factors in a general linear mixed effects model assessing effects of season, segment, position and their interactions on % tensile loss/day. Grey scale from light to dark represents % contribution of season, segment, position (segment), segment x season, position (segment) × season, and error

Percent contribution calculations revealed that season explained the majority of variation (58%) in tensile loss in the studied stream network (Fig. 5). In contrast, location related factors of segment and position cumulatively explained just under 20% of the variation with position explaining just under 2% more variation than segment. Interaction terms cumulatively explained just over 12% of the variation with the position and season interaction explaining about 7% more variation than the segment and season interaction. 11% of the total variation was statistically unexplained.

### Environmental drivers of spatio-temporal patterns of cellulose decomposition

**Fig. 6:**
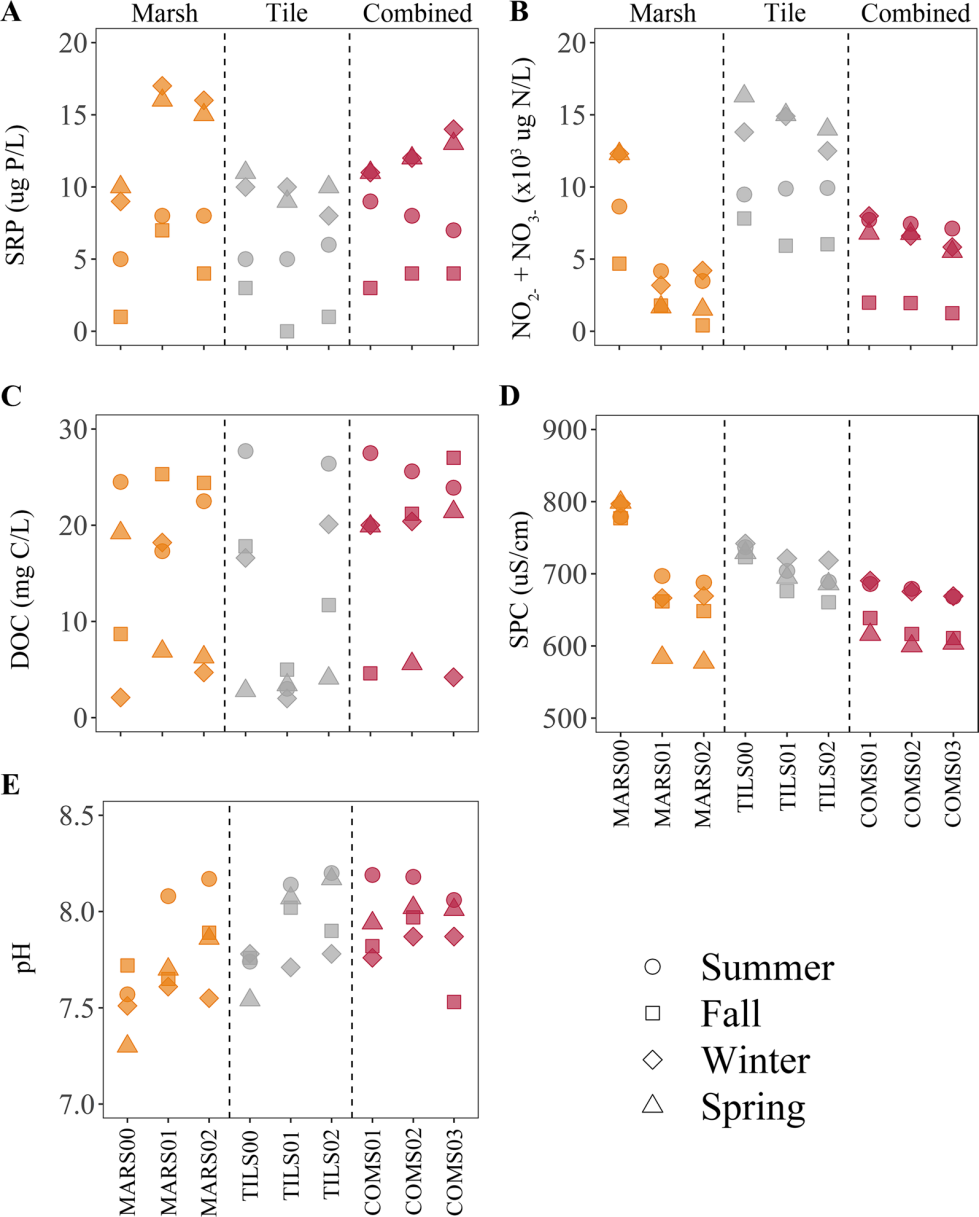
Dot plots showing measured values of soluble reactive phosphorus (SRP; **A**), nitrate-nitrite (**B**), dissolved organic carbon (DOC; **C**), specific conductivity (SPC; **D**), and pH (**E**) at the top, middle, and bottom positions of three segments sourced from the marsh (orange), agricultural tile drain (grey), and downstream trunk (magenta) comprising a headwater stream branch in southwestern Ontario, Canada. Samples collected in summer and fall of 2020 as well as winter and spring of 2021

Measures of bioavailable nutrient forms, SRP, Nitrate-Nitrite and DOC varied across the network of sampled sites (Fig. 6). SRP concentrations typically exceeded 9 µg P/L at all sites in the winter and spring seasons, but only exceeded 5 µg P/L in the autumn season at the middle position of the marsh segment (SRP = 7 µg P/L). Maximum SRP occurred at the middle of the marsh segment (17 µg P/L) in the winter, whereas minimum SRP occurred at the middle of the tile segment (below detection limit) in the fall. Nitrate-nitrite concentrations showed distinct patterns among the segments. In the marsh segment concentrations of nitrate-nitrite varied between 4.7 and 12.3 mg N/L at the top site, but did not exceed 4.2 mg N/L at the lower sites. In contrast, the tile segment had nitrate-nitrite concentrations that ranged over more than 8.0 mg N/L at all sites, with concentrations in winter and spring being at least 4.0 mg N/L greater than those in autumn. The combined segment sites had concentration of nitrate-nitrite between 5.5 and 8.0 mg N/L at all sites for the summer, winter and spring seasons, but concentrations in the fall were all below 2.0 mg N/L. DOC concentrations ranged over more than 18 mg C/L at all sites but the middle site on the tile segment which only ranged between 2 and 5 mg C/L. There was also no apparent season pattern in the ranking of DOC samples, although maximum concentrations mostly came from the summer or autumn season.

Specific conductivity (SPC) and pH showed limited within site variation throughout the study period (Fig. 6). Indeed, SPC ranged less than about 100 µS/cm within all sites. Moreover, SPC measurements ranged less than 100 µS/cm for all sites and seasons in the tile and combined segments. The exception was the Marsh segment for which SPC were more than 100 µS/cm greater at the top site regardless of the season. pH ranged from only 7.3 to 8.2 for all sites and seasons. Within site ranges were even smaller and did not exceed 0.65 at any of the nine sites. There were no clear seasonal patterns in pH, although 7 of the 9 sites exhibited the maximum pH in summer.

**Fig. 7:**
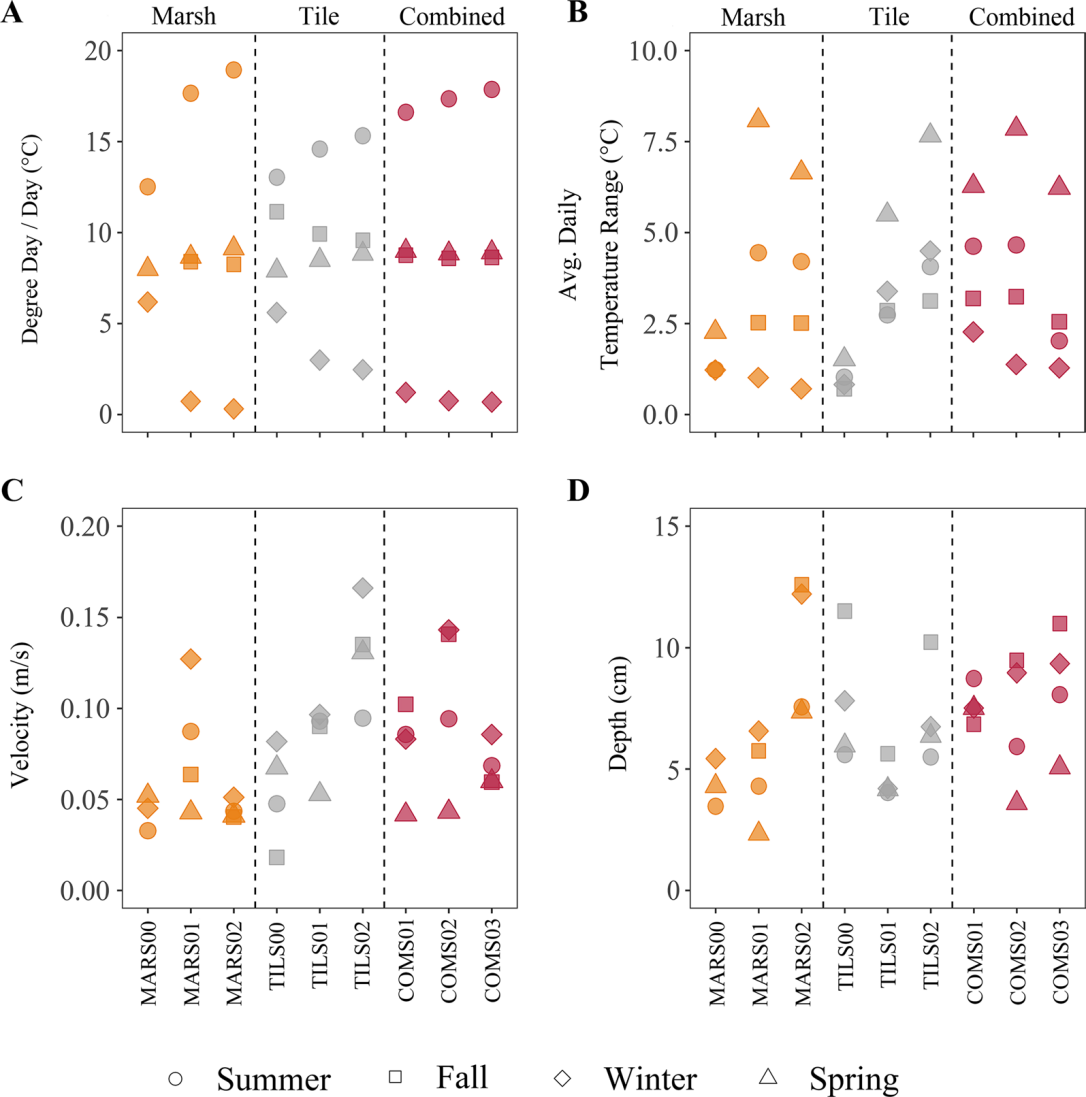
Dot plots showing measured values of degree days/day (**A**) and average daily temperature range (**B**), as well as averaged instantaneous measures of stream velocity (**C**) and stream depth (**D**) above cotton strips deployed at the top, middle, and bottom positions of three segments (marsh (orange), tile (grey), and combined (magenta)) comprising a headwater stream branch in southwestern Ontario, Canada. Samples collected in summer and fall of 2020 as well as winter and spring of 2021

Temperature measures showed variation both in position and season (Fig. 7). Degree day per day were greatest in the summer (> 12.5 °C/day) and lowest in the winter (< 6.2 °C/day) for all sites. Maximum and minimum degree day/day were observed at the bottom of the marsh segment (max: 18.9 °C/day, min: 0.3 °C/day) in the summer and winter, respectively. Average daily stream temperature range was greatest in the spring at all sites (Fig. 6). However, the smallest average daily stream temperature range occurred in the winter in the marsh and combined segment sites, but typically occurred in autumn at the tile sites. The maximum average daily temperature range was observed at the middle position of the marsh segment (8.1 °C) in the spring, whereas the minimum temperature range was observed at bottom of the marsh segment in the winter, and the top of the tile segment in autumn (0.7 °C).

Velocities above the cotton strips averaged between 0.02 and 0.17 cm/s across all sites and seasons (Fig. 7). Moreover, average velocities above the cotton strips typically ranged less than 0.06 cm/s among seasons within a site. Average depth of water over the strips ranged from 2.3 to 12.2 cm. Maximum average depths typically occurred in the autumn season for most sites and were greater at the bottom sites for each segment and the combined sites overall.

PLS analysis on percent tensile loss per day resulted in a significant model (*Q*^2^ = 0.590) that contained one component. The component explained 24.5% of the variance of the independent variables (*R*_*X*_^2^) and 67.7% of the dependent variable (*R*_*Y*_^2^). Degree day/day (VIP = 1.98), and pH (VIP = 1.42) were found to be positively associated with tensile loss. Response variable scores showed that sites were clustered by season, where winter sites typically had the smallest rates of tensile loss and summer sites had the largest rates. Sites in the fall and spring were grouped together between the winter and summer season, although fall observations of tensile loss were skewed more to the positive end of the component than were those from spring.

## Discussion

### Comparison to other studies

Rates of cellulose decomposition observed in our study were within the range of values observed in past studies of minimally and least disturbed, temperate forest streams. For example, autumn rates of cellulose decomposition in our streams were in the lower range of those observed in temperate deciduous forests in a global stream study by Tiegs et al. (2019). Likewise, a study by Webb et al. (2019) of predominately forested streams in southern Ontario, Canada observed an average tensile loss of 1.7 ± 1.1%/day across the spring, summer, and fall seasons that was slightly less than the 1.91 ± 0.96%/day average observed in our study for the same seasons. Furthermore, the range of average tensile loss rates in our summer samples (1.12 to 4.27%/day) encapsulated all but the lowest of rates observed in 25 forested streams in northern Michigan, USA, sampled by Tiegs et al. (2013). In contrast, another southern Ontario study observed a median tensile loss in the spring season of nearly twice that observed in our study (2.43%/day and 1.22%/day, respectively), perhaps reflecting the urban nature of many of the streams used in the study (Kielstra et al. 2019).

We found temperature to be the primary driver associated with seasonal differences in cellulose decomposition as tensile loss was fastest in summer when stream temperatures were warmest and slowest in winter when stream temperatures were coldest. This finding is consistent with past year round studies of cellulose decomposition using the cotton strip assay (Clapcott and Barmuta 2010; Griffiths and Tiegs 2016; Li et al. 2020), and metabolic theory in general (Brown et al. 2004). However, unlike these past studies where winter water temperature minimums were between 5 and 8 °C, our study stream had winter water temps consistently below 5 °C, and as low as 0 °C, yet still exhibited substantial cellulose decomposition (0.57–1.15%/day). The observed winter rates of cellulose decomposition were unanticipated given studies have shown limited growth in members of aquatic microbial communities below 5 °C (Koske and Duncan, 1974; Sridhar and Bärlocher 1993). Yet, although our study is unable to determine the mechanism enabling these relatively rapid rates of cellulose decomposition at cold temperatures, it does indicate the potential for significant organic matter processing to occur throughout the year in streams located in cold climates. Additional studies are clearly needed to better understand the controls and overall role of “under ice” organic matter breakdown in cold regions with ice-forming streams.

Although we are unaware of any other multi-season studies of benthic respiration on cotton strips, rates of respiration observed in our study followed similar temporal patterns trends to other seasonal studies of respiration on leaf packs and inorganic substrates (e.g., Bott et al. 1985; Clapcott and Barmuta 2010), with rates being greatest in summer, followed by the fall and spring. However, unlike past studies where summer rates have typically exceeded those in spring and fall by 100% or more (Bott et al. 1985; Clapcott and Barmuta 2010), average rates of respiration differed only slightly among these seasons in our study. As our summer respiration rates are to the upper end of rates observed on cotton strips deployed in streams in nearby Michigan, USA (Tiegs et al. 2013), it seems likely that the lack of strong seasonal variation in our study streams stems from greater than anticipated respiration rates in spring and fall.

### Effect of subsurface drainage on cellulose decomposition and benthic respiration

Our findings suggest that cellulose decomposition in the tile-sourced segment has been significantly impaired by the installation of subsurface drainage systems. Subsurface drainage systems are known to have a variety of impacts on the physico-chemical conditions of receiving stream ecosystems that could have led to reduced cellulose decomposition (Blann et al. 2009). However, unlike many past studies we did not observe any substantive differences in flow regime between the marsh and tile-sourced segments. Likewise, SRP and DOC were not notably different between segments, although differences may have occurred in high flow events not captured in our samples. Nitrate-nitrite concentrations were markedly greater in the tile-sourced segment, which is in line with past studies reporting high concentrations of nitrate in tile drainage water (e.g., Gentry et al. 1998; Drury et al. 2014). However, as observed nitrate concentrations in all segments were consistently over 2 mg N/L it is unlikely that nitrogen was limiting cellulose decomposition.

Rather, it appears the most likely cause of impaired cellulose decomposition was alteration of the thermal regime of the tile-sourced segment. Temperature has been widely reported to be a primary driver of among stream differences in organic matter processing in streams around the globe (Boyero et al. 2011; Tiegs et al. 2019; Woodward et al. 2012), with increased temperatures being associated with more rapid organic matter breakdown (Young et al. 2008). Moreover, agricultural activities have frequently been associated with warming of streams (Allan 2004) and increases in organic matter processing rates (Paul et al. 2006; Niyogi et al. 2003). Yet, within our study system, it appears that subsurface drainage impaired cellulose decomposition in the tile-sourced segment through stream cooling in the warmer months (June through September). The consistently cooler summer temperatures is probably the result of greater capture of groundwater by tiles and reduced surface warming as a result of burial of the segment’s upper reaches. However, as subsurface drainage does not always intersect the groundwater table the stream cooling we observed may be limited to wet landscapes where tiles are draining aquifers.

Subsurface drainage maintained winter stream temperatures nearly 5 °C warmer in the tile compared to marsh-sourced segments. Yet, the increased winter water temperature in the tile-sourced segment did not result in increased cellulose decomposition. Indeed, cellulose decomposition was significantly greater in the marsh-sourced segment throughout the winter despite stream temperatures in this stream being near 0 °C for several weeks. Given that winter cellulose decomposition was greatest at the uppermost site of the marsh-sourced segment where groundwater inputs maintained stream temperatures similarly warm to those in the tile-sourced segment, we hypothesize that temperature is not the only factor influencing cellulose decomposition as a result of the implementation of subsurface drainage. Indeed, the microbial community in the tile-sourced segment may also have been influenced by agrochemicals, such as insecticides, fungicides, and/or antibiotics in livestock manure, that are regularly applied to the cropland that makes up the tile segments upper drainage area. Previous studies have found that microbial litter decomposition was significantly reduced in the presence of agricultural pesticides as a result of declines in microbe abundance and diversity (Rasmussen et al.^2012^; Schäfer et al.^2007^). Likewise, antibiotics commonly used to treat livestock have been linked to reduced bacterial abundance, although impacts on organic matter processing are less clear (Paumelle et al. 2021). However, as our study did not measure these additional contaminants future studies are needed to test this hypothesis.

Our examination of drivers of tensile strength loss showed that in addition to temperature metrics, pH was positively associated with cellulose decomposition. pH has been found to be a predictor of spatial variation in organic matter breakdown in stream ecosystems by several previous studies (e.g. Clivot et al. 2013; Griffith and Perry 1994; Thompson and Bärlocher 1989; Webb et al. 2019). However, other studies finding pH to be a driver of organic matter breakdown had pH ranges greater than 2.0 (Griffith and Perry 1994; Webb et al. 2019). In contrast, the greatest pH range in any given season in our study was 0.6 and most often about 0.2. As daily fluctuations in pH can exceed 0.5 in streams (e.g., Nimick et al. 1998; Jones et al. 2004), it seems unlikely that a pH range of 0.6, or less, would substantially influence organic matter breakdown. Rather, it is more likely that the pH association is an artifact of pH covarying with temperature, as sites with warmer stream temperatures typically also had higher pH.

We observed less variation in cellulose decomposition among sampling positions in the tile segment than in the marsh segment suggesting implementation of subsurface drainage may have homogenized cellulose decomposition along the tile segment. Moreover, ecological homogenization in the tile segment appears to be the result of limited longitudinal variation in temperatures. For example, in summer the longitudinal increase in degree days from upper to lowermost sampling positions was nearly three fold greater in the marsh compared to the tile segment. The difference in longitudinal warming between the two segments may be the combined effect of channel burial and increased capture of groundwater associated with subsurface drainage. Indeed, the open portion of the tile segment is now 130 m shorter and has about five times the discharge at the uppermost point of the open channel compared to the marsh segment. As a result, the water in the tile stream had much less potential to warm before reaching the lower reaches, compared to water in the marsh segment.

The impacts of subsurface drainage on cellulose decomposition appeared to be transmitted beyond the tile segment through the downstream combined segment. Indeed, temperatures throughout the combined segment were cooler in summer and warmer in winter than the lower two marsh segment locations, suggesting inputs from the tile segment were modulating temperatures hundreds of meters downstream. Moreover, the impact of reduced stream temperature on cellulose decomposition albeit more modest, was significant, particularly in summer. Thus, although the gradual warming and associated increase in cellulose decomposition along the combined segment indicates that subsurface drainage effects may not have perpetuated much further down the network, it does suggest that agricultural drainage systems may be influencing cellulose decomposition at larger scales through regulation of stream temperatures. Larger scaled studies are thus needed to better understand the catchment scale impacts of subsurface drainage on cellulose decomposition.

The effect of subsurface drainage on benthic respiration was much less clear than for cellulose decomposition. Indeed, the pattern of between segment differences for respiration was opposite to decomposition as the tile and marsh segments only differed in the earlier spring samplings when stream temperatures were similar. As stream temperatures were not substantively different in spring, it appears that some other controlling factor was resulting in reduced respiration in the tile segment. However, it is not clear from the environmental characteristics we measured what that controlling variable was. In general, it appears that benthic respiration was less sensitive to subsurface drainage than was cellulose decomposition. As such, cellulose decomposition may be the more useful indicator of the effects of subsurface drainage systems, supporting calls to adopt the cotton strip assay as a biomonitoring tool (e.g., Tiegs et al. 2013; Webb et al. 2019), particularly for the assessment of thermal disturbances resulting from human activities.

### Conclusions and management implications

Our conclusion of impaired cellulose decomposition in the tile stream hinges upon the assumption that the tile and marsh segments were comparable prior to installation of the subsurface drainage system. A lack of pre-development data is a common limitation of environmental assessments of streams in many regions because development occurred several decades or centuries earlier. Some studies attempt to address this lack of pre-development data through space-for-time substitutions where numerous streams are sampled across an exposure gradient (e.g., Grimstead et al. 2018; Stranko et al. 2008; Brown et al. 2009). Yet, space-for-time substitution studies often must trade comparability for replication as even relatively small differences in landscape properties, such as topography, geology and agricultural practices, as well as random events, can introduce confounding variation (Pickett 1989; Damgaard 2019). In our study, we chose to maximize comparability between the marsh (i.e., control) and tile (i.e., treatment) segments by limiting our assessment to two adjoining headwater segments whose close proximity reduced the potential for introduction of significant natural variation. Indeed, the strong correspondence between the seasonal thermal patterns of the upper sites of the marsh and tile segments lends confidence to the assumption that the spring-fed origins and gradual downstream warming observed in the marsh-sourced segment was the pre-development condition in the tile-sourced segment. However, the limited spatial scope of our study means that further studies are required across a range of streams exposed to subsurface drainage to test the universality of the observed relationships between subsurface drainage and cellulose decomposition.

We foresee two immediate implications of our findings to stream conservation and management strategies. First, our study has indicated that subsurface drainage reduced cellulose decomposition by nearly one third in the tile segment and by about half that amount in the downstream combined segment. Given that similar drainage systems occur throughout much of southern Ontario, and in many agricultural regions around the world, coarse extrapolation of our results suggests that many stream networks in agroecosystems may have lost significant capacity to process organic matter. Widespread declines in organic matter processing have implications for aquatic food webs and carbon cycling and may help to explain widespread degradation of aquatic communities in agricultural regions. Second, it is possible that subsurface drainage systems that drain near surface aquifers may provide resiliency to agricultural streams in the face of climate change by inadvertently buffering predicted effects of a warming climate on organic matter processing. However, benefits of cooling may be countered by potential negative effects of increased nutrient and contaminant loadings often associated with agricultural drainage water. Further research needs to be undertaken at larger scales to more fully establish the relationships between subsurface drainage systems and cellulose decomposition and thereby better inform management actions.

## Data Availability

The datasets used and/or analysed during the current study are available from the corresponding author on reasonable request.

## Abbreviations

CSA: Cotton strip assay
DO: Dissolved oxygen
DOC: Dissolved organic carbon
GLM: General linear model
GLMM: Generalized linear mixed-effects model
PLS: Partial least squares
SRP: Soluble reactive phosphorus
SPC: Specific conductivity
